# Chemokine CXCL13 as a New Systemic Biomarker for B-Cell Involvement in Acute T Cell-Mediated Kidney Allograft Rejection

**DOI:** 10.3390/ijms20102552

**Published:** 2019-05-24

**Authors:** Lena Schiffer, Flavia Wiehler, Jan Hinrich Bräsen, Wilfried Gwinner, Robert Greite, Kirill Kreimann, Anja Thorenz, Katja Derlin, Beina Teng, Song Rong, Sibylle von Vietinghoff, Hermann Haller, Michael Mengel, Lars Pape, Christian Lerch, Mario Schiffer, Faikah Gueler

**Affiliations:** 1 Nephrology, Hannover Medical School, 30625 Hannover, Germany; Schiffer.Lena@mh-hannover.de (L.S.); Wiehler.Flavia@mh-hannover.de (F.W.); Gwinner.Wilfried@mh-hannover.de (W.G.); Greite.Robert@mh-hannover.de (R.G.); Kirill.Kreimann@stud.mh-hannover.de (K.K.); athorenz@gmx.de (A.T.); teng.beina@mh-hannover.de (B.T.); rong.song@mh-hannover.de (S.R.); vonvietinghoff.sibylle@mh-hannover.de (S.v.V.); haller.hermann@mh-hannover.de (H.H.); Mario.Schiffer@uk-erlangen.de (M.S.); 2 Pediatric Nephrology, Hannover Medical School, 30625 Hannover, Germany; pape.lars@mh-hannover.de (L.P.); Lerch.christian@mh-hannover.de (C.L.); 3 Pathology, Hannover Medical School, 30625 Hannover, Germany; Braesen.Jan@mh-hannover.de; 4 Radiology, Hannover Medical School, 30625 Hannover, Germany; derlin.katja@mh-hannover.de; 5 Laboratory Medicine & Pathology, University of Alberta, Edmonton, AB T6G 2R3, Canada; mmengel@ualberta.ca; 6 Nephrology and Hypertension, University Hospital Erlangen, 91054 Erlangen, Gerrmany

**Keywords:** B-cell attracting chemokine, CXCL13, kidney transplantation, allograft rejection, T cell-mediated rejection

## Abstract

The presence of B-cell clusters in allogenic T cell-mediated rejection (TCMR) of kidney allografts is linked to more severe disease entities. In this study we characterized B-cell infiltrates in patients with TCMR and examined the role of serum CXCL-13 in these patients and experimentally. CXCL-13 serum levels were analyzed in 73 kidney allograft recipients at the time of allograft biopsy. In addition, four patients were evaluated for CXCL13 levels during the first week after transplantation. ELISA was done to measure CXCL-13 serum levels. For further mechanistic understanding, a translational allogenic kidney transplant (ktx) mouse model for TCMR was studied in BalbC recipients of fully mismatched transplants with C57BL/6 donor kidneys. CXCL-13 serum levels were measured longitudinally, CD20 and CD3 composition and CXCL13 mRNA in tissue were examined by flow cytometry and kidneys were examined by histology and immunohistochemistry. We found significantly higher serum levels of the B-cell chemoattractant CXCL13 in patients with TCMR compared to controls and patients with borderline TCMR. Moreover, in patients with acute rejection within the first week after ktx, a >5-fold CXCL13 increase was measured and correlated with B-cell infiltrates in the biopsies. In line with the clinical findings, TCMR in mice correlated with increased systemic serum-CXCL13 levels. Moreover, renal allografts had significantly higher CXCL13 mRNA expression than isogenic controls and showed interstitial CD20+ B-cell clusters and CD3+ cell infiltrates accumulating in the vicinity of renal vessels. CXCL13 blood levels correlate with B-cell involvement in TCMR and might help to identify patients at risk of a more severe clinical course of rejection.

## 1. Introduction

Kidney allograft rejection is the major cause for loss of graft function and may have a negative impact on long-term allograft survival. Currently, besides donor specific antibodies or non-HLA antibodies, which are linked to humoral rejection, no reliable serum markers for ktx rejection exist [1,2]. Impaired allograft function with elevated serum creatinine drives the decision to perform a biopsy. The majority (about 90%) of acute rejections, especially in the first year after ktx, are T cell-mediated [3]. However, emerging evidence has revealed that intra-graft B-cell accumulation plays an important role in T cell-mediated rejection as well and correlates with a worse outcome [4,5,6]. However, the mechanistic details are not completely resolved. The chemokine CXC ligand 13 (CXCL13), also known as B-cell-attracting chemokine-1 (BAC-1) or B-lymphocyte-chemoattractant (BLC), is a CXC subtype member of the chemokine superfamily. CXCL13 is particularly important in the context of leukocyte recruitment and is sufficient to induce secondary lymphoid nodes [7,8]. Besides B-cell attraction CXCL13 activates different intracellular pathways that are involved in cell survival, invasion and growth and is able to stimulate resident kidney cells to produce pro-inflammatory cytokines and chemokines [7,9,10,11]. In sum, these biological functions of CXCL13 have led us to hypothesize that CXCL13 may also be linked to B-cell accumulation in kidney allografts in the presence of TCMR. 

Here, we were able to show that CXCL13 can be monitored systemically and is elevated in TCMR compared to borderline rejection and controls. In addition, increasing CXCL13 values in the first week after ktx were indicators for acute rejection with B-cell rich infiltrates in two more patients. Furthermore, in a murine model for TCMR after ktx with similar histological TCMR patterns as in patients, we verified systemic upregulation of CXCL13 in blood as well as on an mRNA-level in the allografts. Taken together, our data indicate that CXCL13 blood levels can function as a readily available biomarker for B-cell involvement in T cell-mediated rejection and possibly as a therapeutic target. 

## 2. Results

### 2.1. B-cell Involvement in Kidney Allograft Rejection in Patients 

**Figure 1:**
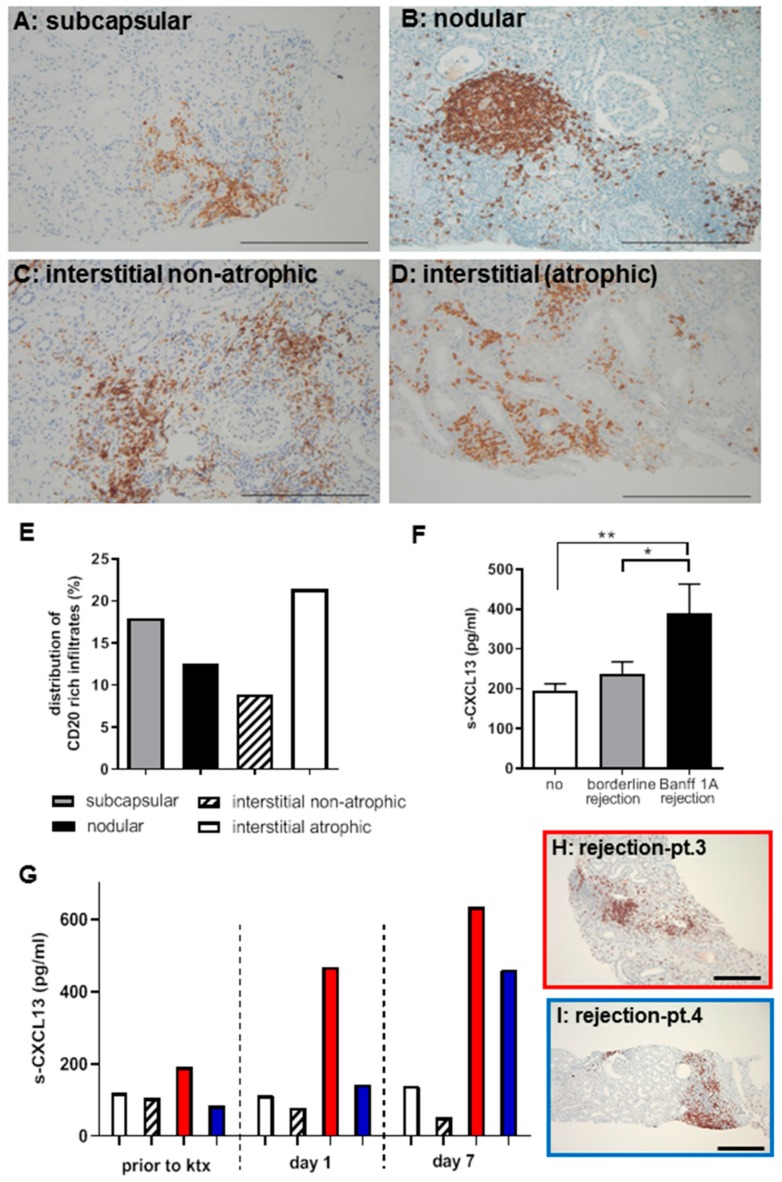
CD20+ cells were detected as part of inflammatory infiltrates in patient biopsies with TCMR (**A**–**D**, bar: 100 μm)) in subcapsular, tubular-interstitial (atrophic and non-atrophic) areas as well as in nodular infiltrates. In the non-rejection state, no CD20 positivity is detectable (data not shown). CD20+ cells were quantified in 67 randomly selected human biopsies with TCMR and graded as B-cell rich (>30 CD20-positive cells/hpf). In subcapsular infiltrates, 17.9%, in interstitial-nodular infiltrates, 12.5%, and in interstitial/atrophic areas, 21.4% were B-cell rich. The Banff-relevant interstitial non-atrophic areas contained 8.9% B-cell rich infiltrates (**E**). Serum CXCL13 levels are increased in patients with TCMR (Banff1a) compared to patients with borderline or no rejection (** *p* = 0.01; * *p* = 0.05) (**F**). Four patients had CXCL13 measurements during the first week after ktx (**G**). All had low levels of CXCL13 prior to ktx and two patients developed a relevant increase of CXCL13 levels up to day 7. Biopsy revealed a rejection with B-cell rich infiltrates (**H**,**I**, bar: 200 μm).

Different morphologies have been described for renal allograft rejection. We characterized different B-cell expression patterns (Figure 1A–E) in 67 randomly selected human biopsies with TCMR and graded them as B-cell rich infiltrates if more than 30 CD20+ cells were detected per high power field (HPF) (Figure 1E).

We found CD20+ rich infiltrates in 83.6% (*n* = 56) of the biopsies while only 16.4% were negative for CD20. The B-cell infiltrates were localized in the subcapsular region in 28.6% of renal biopsies, of these 17.9% contained more than 30 CD20+ cells per HPF. In 19.6% of the biopsies, interstitial-nodular infiltrates occurred, and 12.5% contained more than 30 CD20+ cells per HPF. The majority of CD20+ infiltrates was detected in interstitial/atrophic areas of biopsies (60.7%), more than 30 CD20+ cells per HPF were detected in 21.4% of the biopsies. Interestingly, the nodular infiltrates showed starry sky macrophages and signs of tertiary lymphoid organ formation (TLO) as depicted by CD3 and CD68-stains (Figure S1).

### 2.2. CXCL13 as a Systemic Biomarker of TCMR Rejection in Patients

Serum samples of 73 patients undergoing kidney transplant biopsies with an interval of 39–214 days after surgery (*n* = 28 with allograft rejection and *n*= 45 without rejection) were analyzed by ELISA for systemic CXCL13 expression (Figure 1F). Patient characteristics are summarized in Table 1. Serum CXCL13 was significantly higher in patients with TCMR than in the control group (rejection (*n* = 10 samples); mean 357.6 ±73.2 pg/mL versus controls (*n* = 65 samples; 211.4 ± 19 pg/mL, *p* = 0.006) and also higher compared to patients with borderline rejection (*n*= 24 samples; CXCL13: 214.2 ± 33.7 pg/mL, *p* = 0.06). No significant difference was observed between patients without rejection compared to borderline rejection. In four patients CXCL13 levels were measured longitudinally prior to ktx, at day 1 and 7 after surgery (Figure 1G). All patients had low initial CXCL13 levels and only the two patients with allograft rejection with B-cell rich infiltrates (Figure 1H,J) had >5-fold CXCL13 elevation. Allograft biopsy was indicated when serum-creatinine levels stayed high. Serum-creatinine decreased after anti-rejection therapy (serum-creatinine levels are shown in Figure S2).

### 2.3. Allograft Rejection in a Translational Mouse Model for TCMR

**Figure 2:**
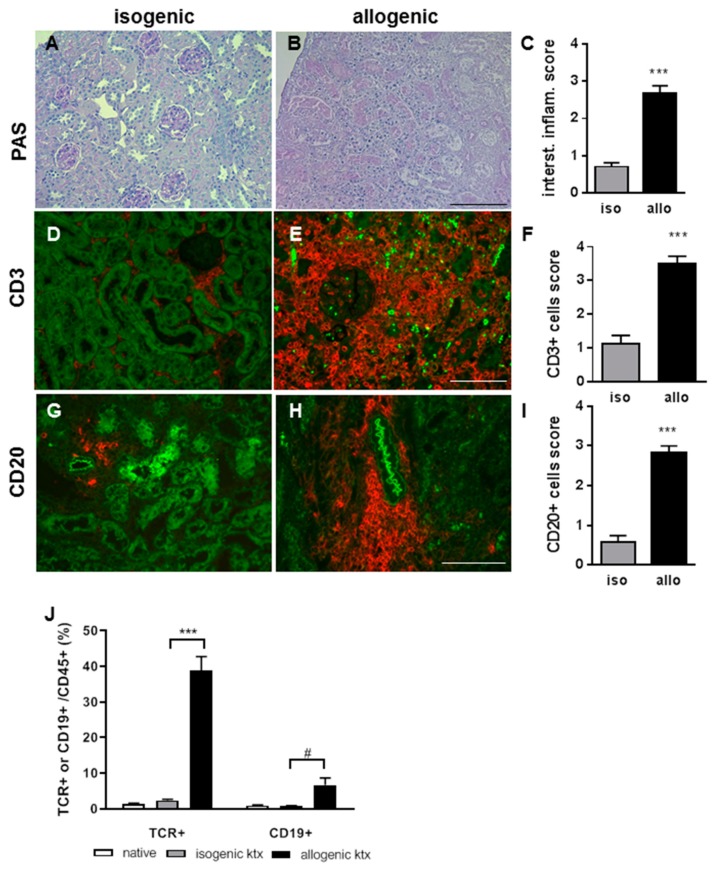
At three weeks after ktx, renal allograft rejection was characterized by severe inflammation and Banff 1A, and higher rejection grades in comparison to isogenic grafts without inflammation (**A**–**C**, representative PAS stains, bar: 200 μm). The majority of infiltration cells were CD3+ T-lymphocytes, which formed interstitial dense infiltrates and clustered around vessels and glomeruli (**D**–**F**, bar: 200 μm). CD20+ nodular B-cell clusters were identified with much higher cell count in allografts compared to isografts (**G**–**I**, bar: 100 μm). Flow cytometry of the infiltrating leukocytes of the grafts showed significantly enhanced proportion of CD3+ T-lymphocytes in allografts and also enhanced CD20+ B-lymphocytes (**J**, *** *p* < 0.001, ^#^ *p* < 0.05).

To study CXCL13 up-regulation in more detail, we investigated a well described translational mouse model of kidney transplantation. The fully mismatched B6 to BALB/c ktx resulted in TCMR and was compared to isogenic ktx (Figure 2). 

**Figure 3:**
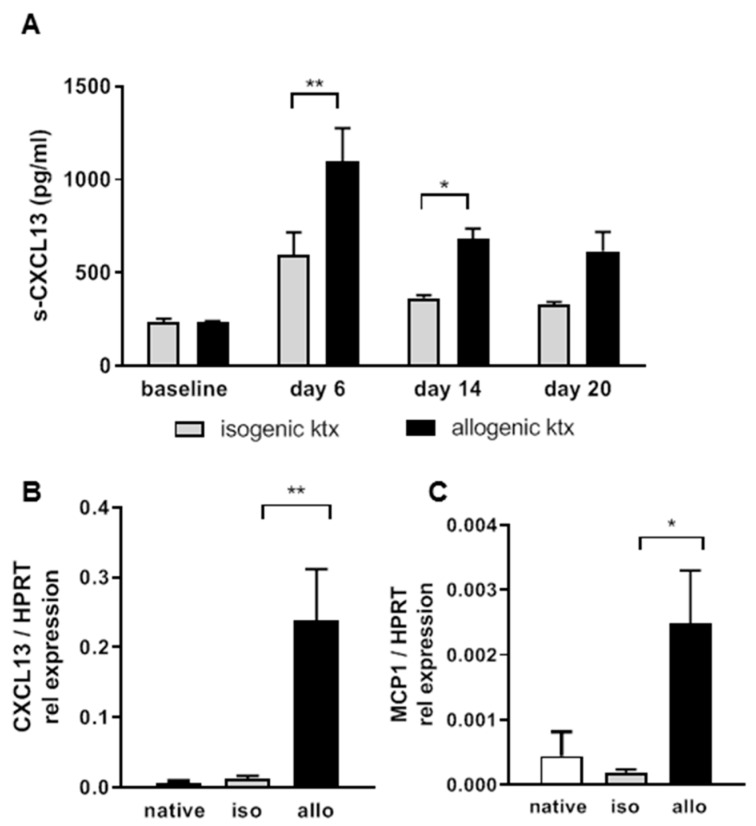
In the mouse ktx model, allograft recipients showed significantly increased serum CXCL13 levels towards day 6 and 14 (**A**). Rejecting kidney allografts showed significantly enhanced CXCL13 mRNA expression compared to isografts and native controls (**B**). MCP-1 mRNA was significantly up-regulated in rejecting kidneys (**C**, *n* = 6 in each group, HPRT served as house keeper, * *p* < 0.05, ** *p* < 0.01).

Histology at three weeks after transplantation revealed Banff 1A or higher rejection in the allografts which had severe interstitial inflammation (Figure 2A–C). The majority of infiltrating cells were CD3+ (Figure 2D–F). In addition, dense, nodular CD22+ B-cell clusters around the vessels could be identified (Figure 2G–I). By flow cytometry, significantly more CD3+ T-cells and also to a lesser extent CD19+ B-cells were observed in the allografts compared to isografts (Figure 2J). In the spleen no differences between T- and B-lymphocytes from allo- or isografts were observed. When measuring systemic CXCL13 expression, a significant increase at day 6 and 14 after ktx in allograft recipients was detected (Figure 3A). 

This was in line with the increased systemic CXCL13 levels in patients. Furthermore, in the renal tissue, significantly enhanced CXCL13 mRNA expression was detected in allografts compared to isografts (Figure 3B). As expected, the pro-inflammatory cytokine monocyte chemoattractant protein (MCP-1) was also significantly upregulated in allografts compared to isografts (Figure 3C). 

## 3. Discussion

Kidney allograft rejection remains a major complication after transplantation along with the risk of graft loss or limited long term graft survival. The majority of rejection types in the early phase after ktx are TCMR [12]. In this context it is known that a significant proportion of TCMR cases additionally contain relevant number of B-cells in the infiltrates [13]. B-cell involvement has been linked to more severe clinical courses of allograft rejection, which were more difficult to control [13,14]. It is generally accepted that B-cells act as positive mediators of inflammation through their production of immunoglobulins and of cytokines such as IL-4 and IL-6. B-cells also support T cell activation by acting as antigen presenting cells and exacerbate renal damage through this interaction [15]. Even though the pathophysiological role is not completely resolved, the association of B-cell infiltrates and an inferior ktx outcome is beyond controversy: different clinical studies have confirmed an association of B-cell infiltrates and a worse transplant survival rate [16,17]. 

Previously, Steinmetz et al. described an association between CXCL13 expression and CXCR5+ and CD 20+ B-cells in acute renal transplant rejection in patients [18]. Interestingly, in the current study we were able to show that B-cells can be detected in the majority of cases with TCMR in patients. We demonstrated that B-cells were, besides localizations in the subcapsular region and the interstitium, mainly located in interstitial non-atrophic areas. Of note, the revised Banff-classification newly introduced the inflammation-interstitial fibrosis and tubular atrophy (i-IFTA) score, which recognizes inflammation within areas of fibrosis and atrophy as relevant [19]. 

Previously, we found that the expression of the B-cell attracting chemokine CXCL13 plays a pivotal role in patients with different B-cell mediated diseases [20]. Besides its role in B-cell homeostasis, the CXCL13/CXCR5 interaction also leads to an activation of different important intracellular pathways such as PI3K/AKT, Raf/MEK/ERK, Integrin-beta3/Scr/FAK and DOCK/Rac/JNK [7,9,10]. These pathways are involved in cell survival, invasion and growth, underlining a more complex function of CXCL13 than initially suspected. In addition, we were able to show that the chemokine CXCL13 was able to stimulate resident kidney cells to produce pro-inflammatory cytokines and chemokines [21]. These mediators lead to a neutrophil respiratory burst, indicating that CXCL13 aggravated the course of disease [21]. 

In the current study, we demonstrated that CXCL13 was significantly increased in the serum of patients with an acute transplant rejection. Of clinical interest, a statistically significant increase was only detected in patients with a clinically relevant rejection, but not in patients with borderline rejection [22]. In a pilot study with four patients we showed that prior to ktx, serum CXCL13 levels were low and increased due to rejection in two patients. A clinical trial is ongoing to validate CXCL13 as a biomarker for early B-cell activation and to study its relevance in the context with allograft rejection shortly after transplantation. Especially in times of organ shortage, this is of clinical interest since allograft rejections are one of the main reasons for transplant loss and early identification of patients at risk allows for early medical intervention.

The sources of CXCL13 production are not completely understood. So far, a variety of transient and non-transient cells have been shown to express CXCL13 [21,23]. Steinmetz et al. showed that CXCL13 expression is exclusively present in areas of B-cell clusters [18]. 

To further investigate the role of CXCL13 and B-cells in acute allograft rejection we used a well-established translational ktx mouse model of TCMR [22]. By using a non-life supporting model where one native kidney remained in situ, we were able to perform longitudinal follow up for three weeks despite ongoing TCMR. In this model, TCMR onset starts at 4–5 days after ktx and is triggered by a prolonged cold ischemia time of 60 min. Flow cytometry and immunohistochemistry showed that the main infiltrating cell type was CD3+ T-lymphocytes. However, similar to the findings in human biopsies, nodular B-cell clusters were also identified in rejecting renal allograft specimens in mice. Accordingly, enhanced CD19+ B-cell population were also identified by flow cytometry in the allografts. Interestingly, the circulating CD19+ B-cell population was reduced in allograft recipients compared to the isogenic controls. This might be explained by the fact that the B-cells were recruited into the lymphoid organs and the rejecting allograft where they form tertiary lymphoid organs [24].

Taken together, our data show that CXCL13 could serve as a relevant circulating biomarker to identify B-cell involvement in ktx recipients with TCMR and relevant B-cell involvement. CXCL13 serum levels may be a readily available surrogate marker for ktx rejection and it is tempting to speculate that CXCL13 could function as a potential therapeutic target.

## 4. Materials and Methods 

### 4.1. Patient Samples

The first clinical study (2001 and 2006) was approved by the Institutional Review Board of Hannover Medical School (approval number 2765). Following informed consent, serum samples from 73 patients were collected at the time of a kidney graft biopsy. Samples for 28 patients with allograft rejection (9 patients with Bannf1A or higher and 19 with Borderline rejection) and 45 patients without rejection were collected. Some patients had repeated biopsies over time so that total sample number was (*n* = 99) (see Table 1). In addition, in an ongoing clinical study (approval number 6895) with longitudinal blood sampling prior to ktx, at day 1 and 7 after ktx we identified two patients with early rejection and two with initial function, and tested for CXCL13 levels in correlation to ktx biopsy (*n* = 4, patient characteristics are shown in Table 2). All patients had standard triple therapy with prednisolone, MMF and tacrolimus and induction therapy with basiliximab. Patient 2 had high panel reactive antibodies (77%) and received plasmapheresis at the day of ktx and afterwards. All serum samples were stored at −80°C.

### 4.2. CXCL13 ELISA

CXCL13 serum levels in human and mice samples were analyzed by ELISA (Quantikine Human CXCL13/BLC/BCA-1 Immunoassay Catalog Number DCX130, Quantikine Mouse CXCL13/BLC/BCA-1, Immunoassay Catalog Number MCX130), respectively, as described previously [10,20]. Color development was measured by using an ELISA reader (Tecan spectra mini, Crailsheim, Germany). The color intensity was correlated with the amounts of bound CXCL13 by comparison with internal standards. 

### 4.3. Renal Morphology and Immunohistochemistry of Patient Allograft Biopsies

Human kidney transplant biopsies were fixed in 4% neutral buffered formaldehyde and embedded in paraffin. Two μm sections were stained for routine histochemical diagnostics (H&E, PAS, Jones Methenamine) according to standard protocols, immunohistochemical stains were performed for CD20 (Dako, clone L26, 1:500 after heat pretreatment with EDTA at pH8.4 for 16 minutes) on an automated platform (Ventana Benchmark Ultra). Normal biopsies can contain single B-cell infiltrates without any clinical relevance. Therefore, we developed a cutoff of >30 CD20+ cells per hpf for definition of relevant “B-cell rich” infiltrates. For the evaluation we used a visual analog scale that we have developed ourselves for standardized evaluation of the samples Figure S3).

### 4.4. Mice

Male C57Bl/6N (B6, H2b) and BALB/cAnCrl (H2d) mice (Charles River, Sulzfeld Germany) weighing 25–28 g at 10–12 weeks of age were used for all experiments. B6 mice served as donors, BALB/c mice as recipients.

### 4.5. Kidney Transplantation

For allogenic kidney transplantation (ktx), B6 mice served as donors and BALB/c as recipients and for isogenic controls, B6 mice served as donors and recipients. Surgeries were done in general anesthesia with isoflurane (induction 3–5% and maintenance 1.5%) by a vascular surgeon with >15 years’ experience in small animal surgery. For analgesia, butorphanol (2.5 mg/kg bodyweight ip) was given prior to surgery [25]. Briefly, the kidney graft was retrieved en bloc with the renal vessels and the ureter. After left recipient nephrectomy, the kidney graft was transplanted. Vessel anastomosis with the abdominal aorta and the caval vene was done. Afterwards, the ureter was directly anastomosed into the bladder dome. Cold ischemia time was standardized to 60 min and warm ischemia time to 30 min. The prolonged cold ischemia time is needed to induce 100% allograft rejection. To overcome mortality in the allograft model, a non-live supporting model was chosen and the right native kidney remained in situ to ensure normal renal function throughout the three weeks observation time. The model has been described previously in detail by functional MRI studies [26,27]. After surgery, animals were monitored until fully awake and had free access to a standard diet (Altromin, Lippe) and tap water. Daily monitoring of physical well-being and behavior was done throughout the follow up of three weeks. Criteria for study termination were impaired food uptake, passive behavior and scrubby appearance. Animals were cared for in accordance to the national and international guidelines of animal welfare and animal protection. Ethical approval was given by the local authority (Lower Saxony Ministry for Food and Drug Safety number: 33.9-42502-04-11/0492). 

### 4.6. Renal Morphology and Immunohistochemistry of Mouse Kidney Grafts

For mouse kidney graft histology and immunohistochemistry two μm paraffin sections were analyzed by PAS stain to define the type and degree of rejection according to the Banff classification [28]. The analysis was done without knowledge of animal group assignment by a nephropathologist with >20 years of experience. Immunohistochemistry for CD3+ T-cell (Acris Antibodies GmbH, Herford, Germany) infiltrates was done on paraffin sections. CD22+ B cells (Southern Biotech, Birmingham, AL, USA) were stained on four μm cryosections. Semi-quantitative assessment of the density of CD3+ cell infiltration was done as follows: 0 < 5 cells per view field (VF40x), 1 mild infiltration: 5–10 cells/VF, 2 moderate infiltration: 11–25 cells/VF, 3 severe: 26–50 cells/VF, 4 very severe >50 cells/VF. B-cell clusters were quantified with 0.5: single CD22+ cell, 1: B-cell cluster with 2–9 CD22+ cells, 2: B-cell cluster with 10–20 CD22+ cells, 3: B-cell cluster with >21 CD22+ cells.

### 4.7. Flow Cytometry 

Flow cytometry of the kidney grafts, whole blood and spleen was done to characterize leukocyte subsets as described previously [27]. FACS Canto-I (BD Biosciences, San Jose, CA, USA) was used for all experiments with FACSDiva software version 6·0. Data were analyzed using WinList™ software (Verity Software House, Topsham, ME, USA). After collagen digest of the renal tissue, live death stain was done. Living leukocytes were stained by CD45+ and CD19+ B-lymphocytes and TCR+ T-lymphocytes were gated from CD11b+ cells. Quantification is given in % from CD45+ cell counts.

### 4.8. CXCL13 Expression in Kidney Graft Tissue

After organ retrieval, tissue was immediately fixed in RNAlater. Total mRNA was extracted using the RNeasy mini kit system (Qiagen, Hilden, Germany) and transcribed with Quiagen mini kits. For quantitative PCR (qPCR), 1 μg of DNase-treated total RNA was reverse transcribed using Superscript II Reverse transcriptase (Invitrogen, Carlsbad, CA, USA) and qPCR was performed on a Lightcycler 420 II (Roche Diagnostics, Penzberg, Germany) using FastStart Sybr-Green. Gene-specific primers for CXCL13 (Primer-sequence: fwd-TCT GGA CCA AGA rev-TGA AGA AAG TT) and monocyte chemoattractant protein-1 (MCP-1; Mm_Ccl2_1_SG QuantiTect Primer Assay QT00167832) were used. Quantification was carried out using QGene software.

### 4.9. Statistical Analysis

For human data we used R (Version 3.3.3) for analysis [29]. As some patients were investigated more than once, we had to account for the correlated nature of the data. We used the multgee package, which applies a generalized estimating equations (GEE) approach for correlated multinomial responses using a local odds ratios parameterization [30]. Besides, statistical analysis was performed with GraphPad Prism 6.0 (GraphPad Software, Inc., La Jolla, CA, USA). One-way ANOVA for multiple comparison was used with post-hoc Tukey correction. For comparison of two groups, *T*-test was done. Values are expressed as mean with standard error of mean (SEM). Significance was assumed at * *p* < 0.05, ** *p* < 0.01, *** *p* < 0.001.

## Figures and Tables

**Table 1:** Patient characteristics (study 2001–2006).

Type of Rejection	Banff 1A or Higher	Borderline	No Rejection
Number of patients	9	19	45
Number of serum samples	10	24	65
Time between transplantation and biopsy (days; ±SD)	108.9 (±56.2)	110.4 (±59.0)	117.0 (±53.6)
Age at transplantation (years; ±SD)	54.1 (±19.6)	48.3 (±10.2)	54.5 (±12.6)
HLA-Mismatch (mean ± SD)	1.5 (±2.3)	1.9 (±1.6)	2.1 (±1.7)
Data available for the following number of patients	8 (9)	15 (19)	41 (45)
Creatinine level at time of biopsy (μmol/l; ±SD)	160.1 (±72.9)	194.0 (±91.8)	154.1 (±72.4)
Creatinine level after 1 year (μmol/l; ±SD)	156.4 (±41.1)	188.1 (±73.7)	142.2 (±63.9)
Creatinine level after 5 year (μmol/l; ±SD)	146.2 (±41.9)	196.9 (±91.5)	162.9 (±99.9)
Immunosuppression at time of biopsy			
Number of immunosuppressants (±SD)	2.2 (±0.6)	2.2 (±0.4)	2.6 (±0.6)
Data available for the following number of samples	10 (10)	23 (24)	62(65)
Prednisolon dose (mg; ±SD)	10.5 (±5.7)	12.2 (±5.3)	9.6 (±5.4)
Cyclosporine A	50%	91.7%	88.7%
Mycophenolat mofetil	20%	25%	59.7%
Sirolimus	0%	8.3%	16.1%
Tacrolimus	30%	4.2%	3.2%
Belatacept	20%	0%	4.8%
Number of rejections (mean ± SD)	1.2 (±0.4)	0.2 (±0.4)	0 (±0)
DSA-Status	n.d.	n.d.	n.d.

**Table 2:** Pilot study in the early phase after ktx.

Patient Characteristics	Patient 1	Patient 2	Patient 3	Patient 4
Recipient age (years)	22	48	51	50
Hemodialysis (years)	None	6	12	12
Type of ktx	living donation	postmortal, AM-Program	postmortal	postmortal
Plasmapheresis	no	2× day 0 + 1	day 15, 16, 17	no
Delayed graft function	no	no	no	yes
Allograft biopsy (day)	n/a	n/a	10	14
In hospital stay (days)	8	8	21	14
Steroid boli for rejection treatment	n.a.	n.a.	3× 500 mg prednisolone	3× 500 mg prednisolone
Creatinine at 4 weeks after ktx (μmol/L)	145	178	162	169

## Supplementary Materials

Supplementary materials can be found at https://www.mdpi.com/1422-0067/20/10/2552/s1.

## Abbreviations

BCA	B-cell attracting chemokine
CXCL13	CXC ligand 13 protein
ktx	Kidney transplantation
allograft rejection	T cell-mediated rejection
