# Supplementary material for: Chemokine CXCL13 as a New Systemic Biomarker for B-Cell Involvement in Acute T Cell-Mediated Kidney Allograft Rejection
Source: Int J Mol Sci. 2019 May 24;20(10):2552. doi: 10.3390/ijms20102552 (PMC6567305; doi:10.3390/ijms20102552)
Supplement: Supplementary file 1 [file ijms-20-02552-s001.pdf]

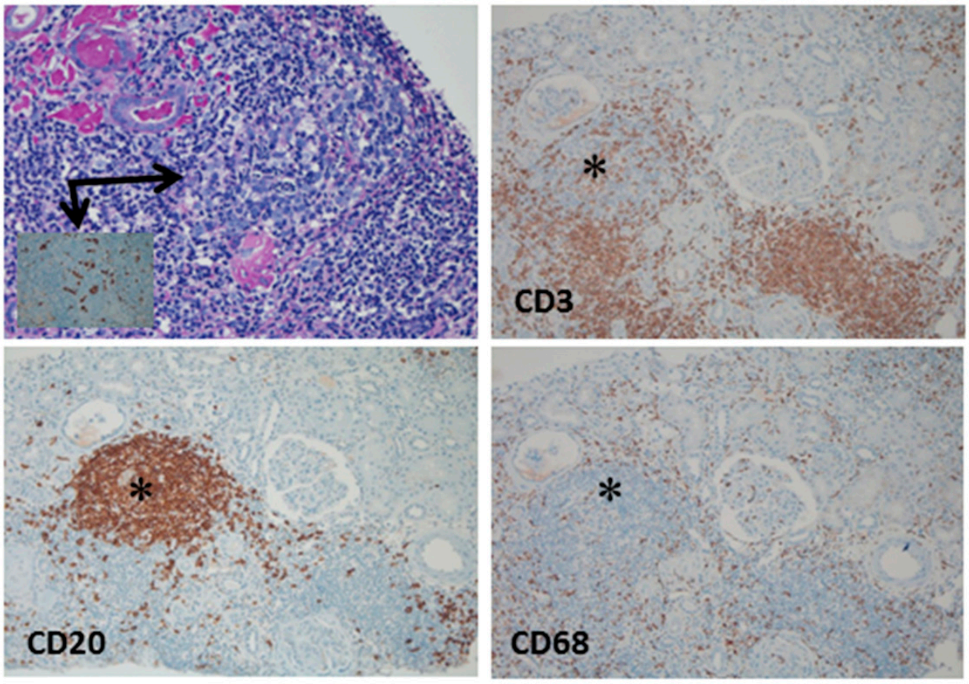

Figure S1: TLO formation can be demonstrated in nodular infiltrate (asterisk) in a rejection biopsy (compare Figure 1A). Upper left: PAS stain with germinal center (arrow), inset and small arrow point out starry sky macrophages (CD68). Same area stained for T-lymphocytes (CD3), B-lymphocytes (CD20) and macrophages (CD68) as labelled. Original magnification: 400x in upper left, 100x in all others.

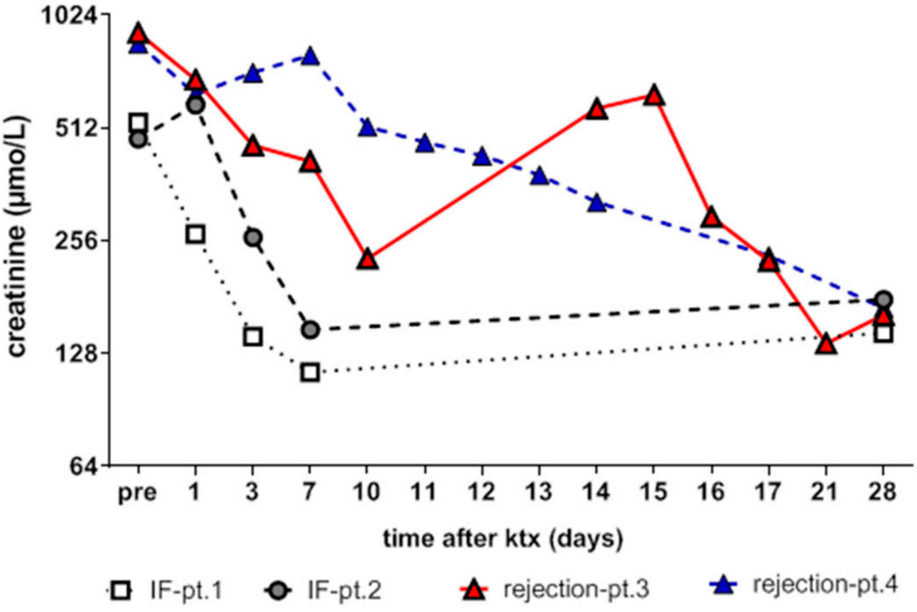

Figure S2: Creatinine follow-up of the four patients in the early phase after transplantation, depicting the normal slope of serum creatinine in patient 1 and 2 vs. creatinine slope in the two rejection patients 3 and 4 (compare Figure 1G,H,I).

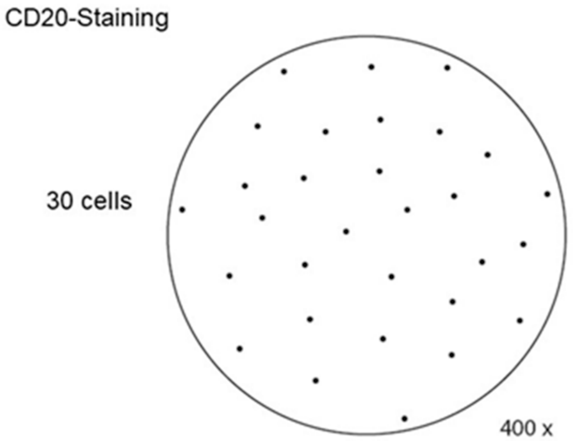

Figure S3: The diagram depicts the visual analog scale for standardized quantification of B-cell numbers in biopsy infiltrates. B-cell rich infiltrates were defined as more than 30 CD20 positive cells per high power field at 400x magnification.
